# Genomic characterization of a *Helicobacter pylori* isolate from a patient with gastric cancer in China

**DOI:** 10.1186/1757-4749-6-5

**Published:** 2014-02-24

**Authors:** Yuanhai You, Lin Liu, Maojun Zhang, Yuanfang Zhu, Lihua He, Dongfang Li, Jianzhong Zhang

**Affiliations:** 1 Collaborative Innovation Center for Diagnosis and Treatment of Infectious Diseases, State Key Laboratory for Infectious Disease Prevention and Control and National Institute for Communicable Disease Control and Prevention, Chinese Center for Disease Control and Prevention, Beijing, People’s Republic of China; 2 BGI-Shenzhen, Shenzhen, People’s Republic of China

**Keywords:** *Helicobacter pylori*, Gastric cancer, Next generation sequencing, Genomic features

## Abstract

**Background:**

*Helicobacter pylori* is well known for its relationship with the occurrence of several severe gastric diseases. The mechanisms of pathogenesis triggered by *H. pylori* are less well known. In this study, we report the genome sequence and genomic characterizations of *H. pylori* strain HLJ039 that was isolated from a patient with gastric cancer in the Chinese province of Heilongjiang, where there is a high incidence of gastric cancer. To investigate potential genomic features that may be involved in pathogenesis of carcinoma, the genome was compared to three previously sequenced genomes in this area.

**Result:**

We obtained 42 contigs with a total length of 1,611,192 bp and predicted 1,687 coding sequences. Compared to strains isolated from gastritis and ulcers in this area, 10 different regions were identified as being unique for HLJ039; they mainly encoded type II restriction-modification enzyme, type II m6A methylase, DNA-cytosine methyltransferase, DNA methylase, and hypothetical proteins. A unique 547-bp fragment sharing 93% identity with a hypothetical protein of *Helicobacter cinaedi* ATCC BAA-847 was not present in any other previous *H. pylori* strains. Phylogenetic analysis based on core genome single nucleotide polymorphisms shows that HLJ039 is defined as hspEAsia subgroup, which belongs to the hpEastAsia group.

**Conclusion:**

DNA methylations, variations of the genomic regions involved in restriction and modification systems, are the “hot” regions that may be related to the mechanism of *H. pylori*-induced gastric cancer. The genome sequence will provide useful information for the deep mining of potential mechanisms related to East Asian gastric cancer.

## Background

*Helicobacter pylori*, a Gram-negative bacterium that colonizes in the human stomach, has been widely recognized as a pathogenic bacteria related to the pathogenesis of gastritis, ulcers, and carcinoma [1-3]. The high genetic variability of *H. pylori* drives its dramatic ability to adapt to the gastric niche [4-9]. However, although many studies have been performed, its mechanisms are still not well elucidated.

With the rapid development of the next generation sequencing technology and reduced costs, it has become possible to perform large scale genome sequencing procedures to obtain ample information about biological population structure and disease markers. Over the past few years, increasingly more *H. pylori* strains from different geographic regions, ethnicities, and diseases have been sequenced [10-12], and at least 50 genome sequences are currently available in public databases.

In a previous study, we published genome sequences of three strains recovered from patients with ulcers and atrophic gastritis in Heilongjiang province [13]. It is well known that *H. pylori* strains isolated from different geographic areas show dramatic genomic diversity [14]. Thus, at the genomic level, comparative analysis among strains with different clinical manifestations should initially eliminate such interference. Comparative genomic sequencing analysis of strains isolated from single patients could be a reliable way to eliminate such interference [15-17]. However, it is usually difficult to follow a patient and obtain strains isolated from various unpredictable manifestations.

In this study, we reported a draft genome sequence of strain HLJ039 that was isolated from a patient with gastric cancer in Heilongjiang province. After integration with the other three genomes from the same area, initial comparative genomic analysis was performed to investigate the genetic features of gastric cancer isolates.

## Methods

### Strain selection

HLJ039 was isolated from an 84-year-old man with poorly differentiated stomach body cancer. Although some other gastric carcinoma-related *H. pylori* strains isolated from different areas, ethnicities, and populations in the world are present in public databases, we did not select these strains for our comparative analysis. The complex strain background will make it very difficult to identify reliable genomic characteristics that may be contributed to a specific disease like gastric cancer. As such, analyzing a specific geographic region, ethnicity, or population may be a more sensible way to find potential clues related to specific diseases. Therefore, in this study, we selected only three strains isolated from Heilongjiang province for the comparative analysis. These strains are very representative because Heilongjiang province has a high incidence of gastric diseases in China, especially for gastric cancer. In addition, the Chinese Heilongjiang province is near Korea and Japan. These east Asian countries reportedly have the highest incidence of gastric cancer worldwide [18,19].

## Ethics approval

This research was approved by the meeting of ethics committee of national institute for communicable disease control and prevention, China CDC, according to Chinese ethics laws and regulations. NO:ICDC-2013001.

### Genome sequencing and annotation

The strain was isolated from gastric mucosa and cultured on Columbia agar base supplemented with 5% sheep blood. DNA was extracted as previously described [20]. For each strain, whole-genome sequencing was performed using an Illumina Hiseq 2000 by generating paired-end libraries (500 bp and 2 kb) following the manufacturer’s instructions. The read lengths were 90 bp and 50 bp for each library, from which more than 100 Mb of high-quality data was generated. The paired-end reads from the two libraries were de novo assembled into scaffolds using SOAPdenovo (http://soap.genomics.org.cn). Gene prediction was performed using Glimmer. The tRNA genes were searched for by tRNAScan-SE2, while the rRNA genes were searched for by RNAmmer3. Protein BLAST4 was run using the translated coding sequences as a query against the reference sequence (*H. pylori* strain 51).

The genome was further annotated and functionally categorized by Rapid Annotation using Subsystem Technology (RAST). A subsystem is a set of functional roles that an annotator has decided are related. Subsystems frequently represent the collection of functional roles that compose a metabolic pathway, complex, or protein class [21].

### Initial comparative genomic and phylogenetic analysis

To identify possible regions that may be involved in the pathogenesis of gastric cancer, MAUVE was used to compare HLJ039 with three additional isolates recovered from the same area [22]. As described previously, HLJ271 was recovered from a patient with gastric ulcer. HLJ193 and HLJ256 were recovered from patients with atrophic gastritis. Different regions (DRs) of HLJ039 were labeled along its chromosome location. DRs refer to coding sequence (CDS) insertion and deletion in HLJ039 compared to the other three genomes.

To define the phylogenetic characterization of HLJ039 using the publicly available *H. pylori* genome sequences, 53 whole genome sequences were extracted from GenBank for phylogenetic tree construction (Additional file 1). P12 was used as a reference genome. Comparisons were made using the nucmer program from MUMMER3 implemented in Panseq [23]. Genomes were fragmented into 500-bp segments that had to be present in all 54 genomes to be included in the core genome. Horizontally transferred genes usually have high genetic diversity among different strains, for example, the plasticity zones, which encode type IV secretion systems, R-M systems, or transferable genomic islands. According to the principle of multiple alignment by the use of Panseq, these potential horizontal genes would be removed from the core genes. Single nucleotide polymorphisms (SNPs) in the core genomes are determined and used to generate a Phylip-formatted file. Concatenated SNPs in length of 29,259-bp were used to construct a phylogenetic tree by using the neighbor-joining method in MEGA5. Bootstrap method was used to assess the stability of the phylogenetic relationships.

### Genomic data deposition

This whole genome shotgun project has been deposited at DDBJ/EMBL/GenBank under accession number JAAA00000000, while version JAAA01000000 is described in this paper.

## Quality assurance

The genomic DNA was extracted from a pure cultured *H. pylori* strain and confirmed using conventional biochemical tests (positive for urease, catalase, and oxidase). The RAST server was used to evaluate potential heterogeneous contaminations.

## Initial findings

**Figure 1:**
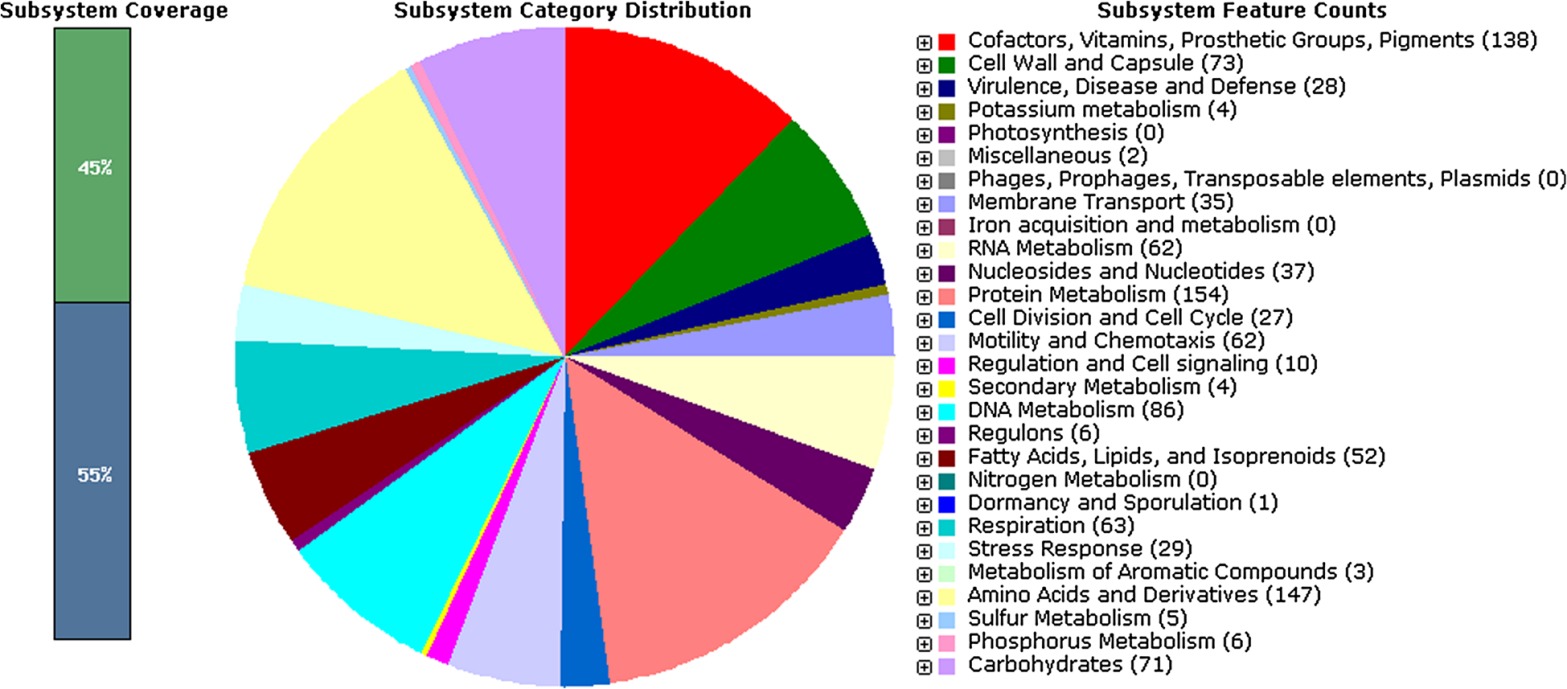
**Subsystem distribution statistics of *Helicobacter pylori *** **strain HLJ039 generated by the rapid annotation using subsystem technology server.**

**Figure 2:**
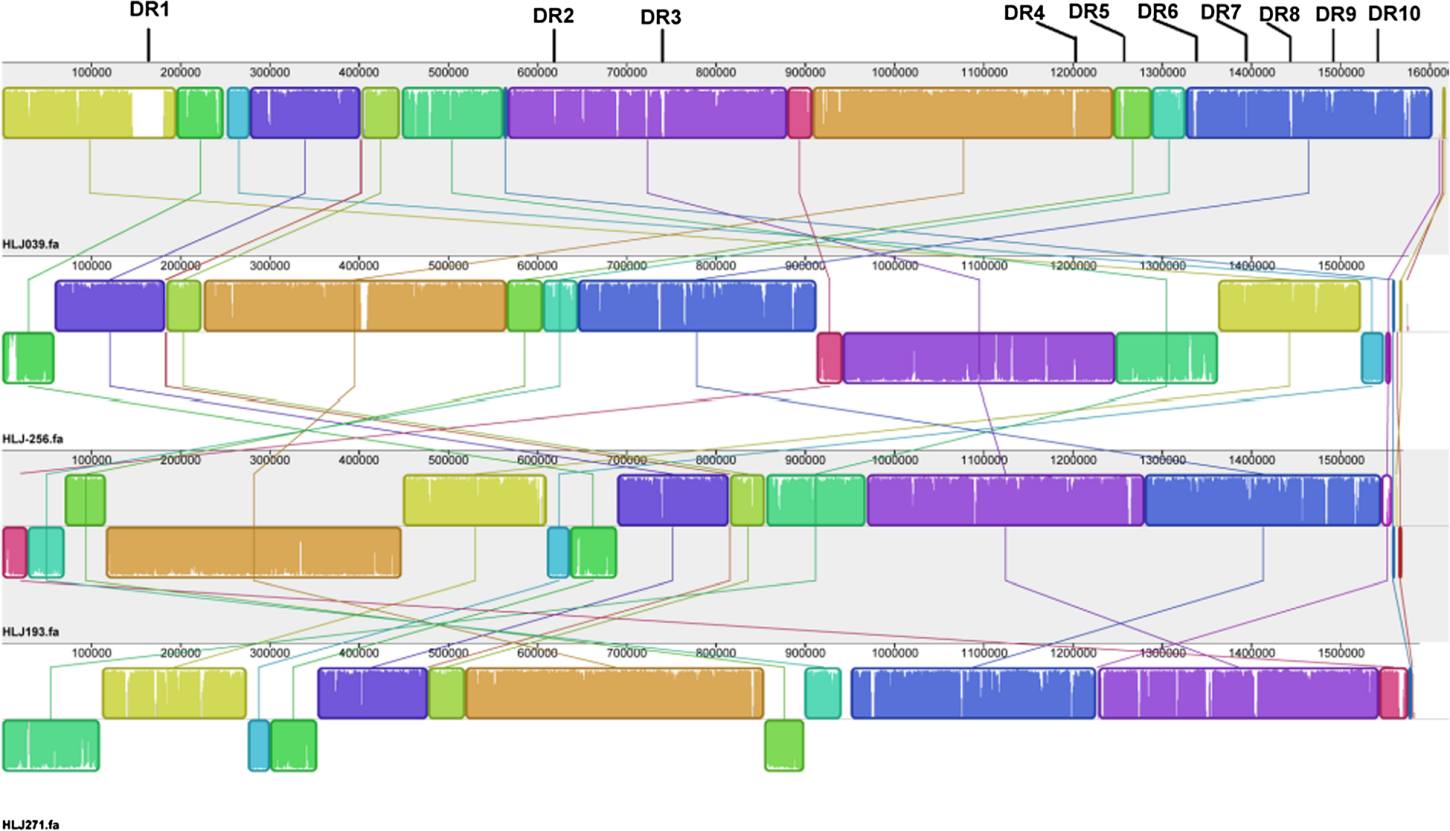
Genome alignment of gastric carcinoma isolate HLJ039 with non-carcinoma isolates.

We ultimately obtained 42 contigs with a total length of 1,611,192 bp and predicted 1,687 CDS within the draft genome of strain HLJ039. Additional information is included in the sequencing reports of HLJ039 (Additional file 2). The G + C content was 38.72%. The subsystem distribution and general information about the potential functional distribution of HLJ039 are shown in Figure 1. Compared to the additional three HLJ genomes, HLJ039 has 10 different regions (DRs). Detailed information about these fragments is shown in Table 1. The locations of these DRs are labeled in the whole genome (Figure 2). Approximately half of these sequences encoded hypothetical proteins. Most of the DR sequences encoded proteins involved in DNA methylase and a restriction modification enzyme. Notably, a unique 547-bp fragment (DR9) sharing 93% identity with a hypothetical protein of *Helicobacter cinaedi* ATCC BAA-847 was found that had never been present in any other *H. pylori* strains previously, which indicated a possible horizontal gene transfer between *H. pylori* and *H. cinaedi*. DR9, located in scaffold 5, inserts into a 1,371-bp gene encoding type III restriction endonuclease, which is responsible for adenine-specific DNA methylase modifications.

**Table 1:** Basic information of the different regions (DRs) in HLJ039

**DR**	**Start**	**End**	**Gene description**
DR1	145736	180926	25 hypothetical proteins,VirB4, DNA topoisomerase I, ParA, Mobile element protein, First ORF in transposon ISC1904
DR2	618752	619703	Fucosyltransferase
DR3	740131	740654	Hypothetical protein
DR4	1200420	1202309	Hypothetical protein
			DNA-cytosine methyltransferase
DR5	1254233	1256053	Hypothetical protein
DR6	1335551	1337398	Type II m6A methylase (hinFIM)
			hypAIVR
DR7	1393932	1394805	Hypothetical protein
DR8	1443251	1445196	Type II DNA modification enzyme
			hypothetical protein
DR9	1484058	1484604	Hypothetical protein sharing 93% identity with a fragment of *Helicobacter cinaedi* ATCC BAA-847
DR10	1538060	1539662	Type IIG restriction and modification enzyme

**Figure 3:**
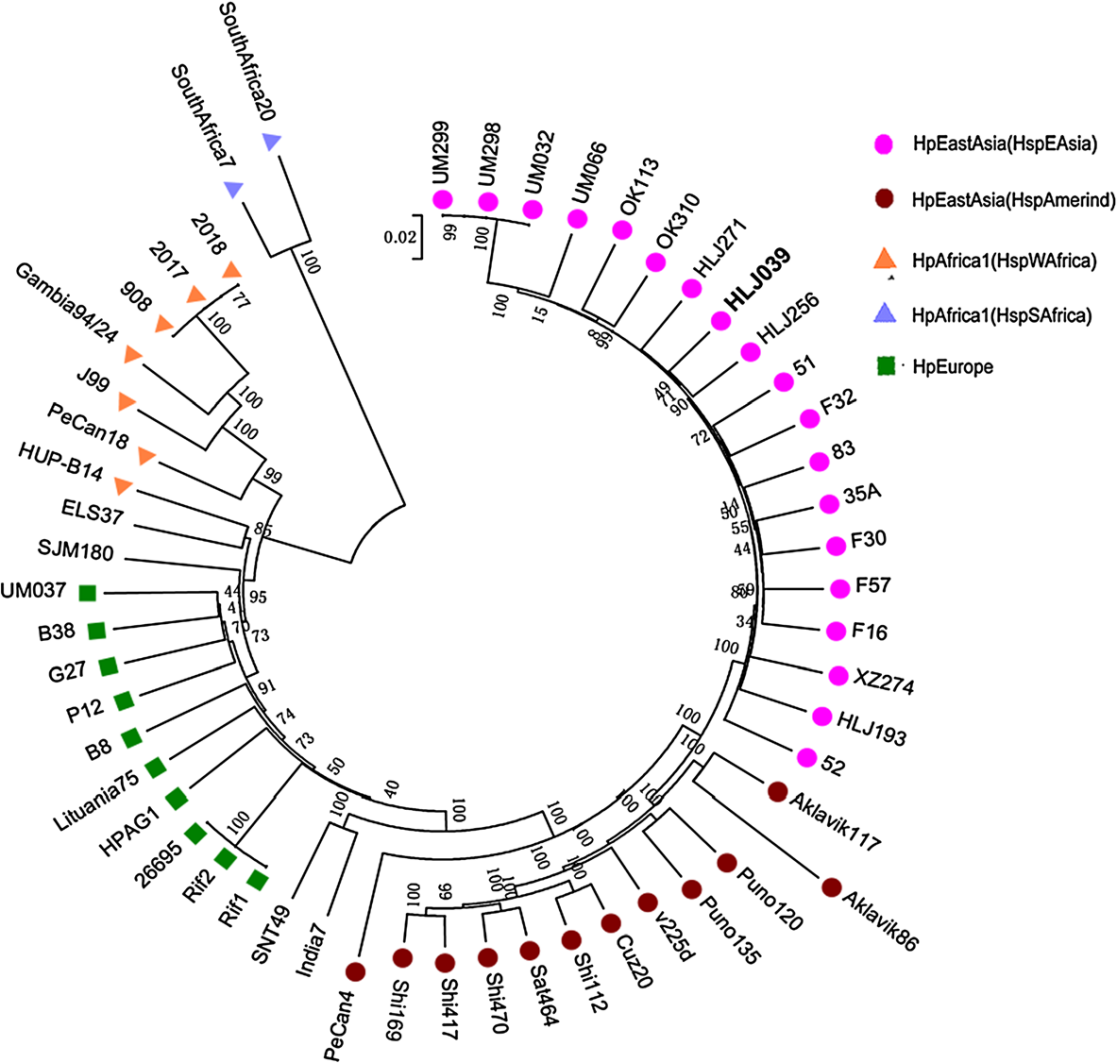
**Phylogenetic analysis of 54 *Helicobacter pylori *** **strains based on their core genome single nucleotide polymorphisms.**

Note: Different regions (DRs) refers to coding sequence insertion and deletion in HLJ039 compared to the other three genomes.

All of the above findings highlight the important role of DNA restriction modification systems in *H. pylori* genomic recombination. A total of 29,259 core SNPs were found among the 54 analyzed genome sequences. Based on a core genome SNP analysis of 54*H. pylori* strains distributed in various worldwide regions, a phylogenetic tree was generated to show the HLJ039 subtype. All strains were classified into different groups defined by earlier studies according to multilocus sequence typing [24,25]. Figure 3 shows that HLJ039 was defined as belonging to the hspEAsia subgroup, which belonged to the hpEastAsia group.

## Future directions

The incidence of gastric carcinoma in East Asian countries is quite high [18,19]. To explore the potential pathogenic mechanisms that may contribute to this phenomenon, more East Asian *H. pylori* strains must first be sequenced. The strains selected for sequencing should be representative and eliminate geographic variation. Our future directions will focus on large-scale genomic sequencing of different clinical isolates from areas with a high incidence of gastric cancer. More detailed analyses involved in DNA methylation as well as restriction and modification systems would be the most attractive directions for studies of *H. pylori*-induced gastric cancer.

### Consent

Written informed consent was obtained from the patient for the publication of this report and any accompanying images.

## Availability of supporting data

Additional data supporting the results reported here are included within the additional files.

## Competing interests

The authors declare that they have no competing interests.

## Authors’ contributions

YY performed the bioinformatics analysis and wrote the manuscript; MZ and LH were responsible for bacteria isolation and identification; LL, XH and YZ performed genomic sequencing; JZ and PN designed the study and provided financial support for this work. All authors read and approved the final manuscript.

## Supplementary Material

Additional file 1 General information for the publicly available genomes. Click here for file

Additional file 2 Assembly information for HLJ039. Click here for file
