# Supplementary material for: Genomic characterization of a Helicobacter pylori isolate from a patient with gastric cancer in China
Source: Gut Pathog. 2014 Feb 24;6:5. doi: 10.1186/1757-4749-6-5 (PMC3938082; doi:10.1186/1757-4749-6-5)
Supplement: Additional file 1 — General information for the publicly available genomes. [file 1757-4749-6-5-S1.doc]

General information for the publically available genomes used in this study.

| Strain | ACCESSION |
| --- | --- |
| 26695 | AE000511 |
| J99 | AE001439 |
| HPAG1 | CP000241 |
| P12 | CP001217 |
| G27 | CP001173 |
| Shi470 | CP001072 |
| Shi169 | NC_017740 |
| Shi417 | NC_017739 |
| Shi112 | NC_017741 |
| 35A | CP002096 |
| 51 | CP000012 |
| 52 | CP001680 |
| 908 | CP002184 |
| B38 | FM991728 |
| B8 | FN598874 |
| Cuz20 | CP002076 |
| Gambia94/24 | CP002332 |
| India7 | CP002331 |
| Lithuania75 | CP002334 |
| PeCan4 | CP002074 |
| Pecan18 | NC_017742 |
| SJM180 | CP002073 |
| Sat464 | CP002071 |
| SouthAfrica7 | CP002336 |
| SouthAfrica20 | NC_022130 |
| v225d | CP001582 |
| 2017 | CP002571 |
| 2018 | CP002572 |
| F16 | AP011940 |
| F30 | AP011941 |
| F32 | AP011943 |
| F57 | AP011945 |
| OK310 | NC_020509 |
| OK113 | NC_020508 |
| UM032 | NC_021215 |
| UM037 | NC_021217 |
| UM066 | NC_021218 |
| UM298 | NC_021882 |
| UM299 | NC_021216 |
| 83 | CP002605 |
| XZ274 | NC_017926 |
| Rif1 | CP003905 |
| Rif2 | CP003906 |
| Puno120 | NC_017378 |
| Puno135 | NC_017379 |
| HUP-B14 | NC_017733 |
| ELS37 | NC_017063 |
| SNT49 | NC_017376 |
| Aklavik86 | NC_019563 |
| Aklavik117 | NC_019560 |
| HLJ039 | JAAA00000000 |
| HLJ256 | ALKA00000000 |
| HLJ271 | ALKB00000000 |
| HLJ193 | ALJI00000000 |
