# Supplementary material for: Genomic characterization of a Helicobacter pylori isolate from a patient with gastric cancer in China
Source: Gut Pathog. 2014 Feb 24;6:5. doi: 10.1186/1757-4749-6-5 (PMC3938082; doi:10.1186/1757-4749-6-5)
Supplement: Additional file 2 — Assembly information for HLJ039. [file 1757-4749-6-5-S2.doc]

Assembly information for HLJ039

| Strain | Clean Data(Mb) | Num of contigs | Total length(bp) | N50(bp) | N90(bp) | G+C% |
| --- | --- | --- | --- | --- | --- | --- |
| HLJ039 | 100 | 42 | 1,611,192 | 176,997 | 39,276 | 38.72 |
